# Retraction Note: *Eobania vermiculata* whole-body muscle extract-loaded chitosan nanoparticles enhanced skin regeneration and decreased pro-inflammatory cytokines in vivo

**DOI:** 10.1186/s12951-025-03296-z

**Published:** 2025-03-14

**Authors:** Alyaa Farid, Adham Ooda, Ahmed Nabil, Areej Nasser, Esraa Ahmed, Fatma Ali, Fatma Mohamed, Habiba Farid, Mai Badran, Mariam Ahmed, Mariam Ibrahim, Mariam Rasmy, Martina Saleeb, Vereena Riad, Yousr Ibrahim, Neveen Madbouly

**Affiliations:** 1 Biotechnology Department, Faculty of Science, Cairo University, Giza, Egypt; 2 Biotechnology/Biomolecular Chemistry Program, Faculty of Science, Cairo University, Giza, Egypt; 3 Zoology Department, Faculty of Science, Cairo University, Giza, Egypt

**Retraction Note: Journal of Nanobiotechnology (2023) 21:373**

10.1186/s12951-023-02143-3

The Editor-in-Chief has retracted this article. After publication, concerns were raised regarding repetitive patterns and similarities in a number of images presented in Fig. 9. Further checks by the Published identified additional image irregularities in Figs. 4B and 8.

The authors provided partial original images for Figs. 4B and 8, which confirmed that the published images were digitally modified beyond the acceptable limits stated in our Editorial policies.

The Editor-in-Chief therefore no longer has confidence in the presented data.

Alyaa Farid does not agree to this retraction. None of the other authors have responded to any correspondence from the publisher about this retraction.

